# An innovative monolithic zwitterionic stationary phase for the separation of phenolic acids in coffee bean extracts by capillary electrochromatography

**DOI:** 10.1016/j.aca.2017.01.048

**Published:** 2017-02-02

**Authors:** Adele Murauer, Rania Bakry, Herwig Schottenberger, Christian Huck, Markus Ganzera

**Affiliations:** a Institute of Pharmacy, Pharmacognosy, University of Innsbruck, Innsbruck, Austria; b Institute for Analytical Chemistry and Radiochemistry, University of Innsbruck, Innsbruck, Austria; c Institute of General, Inorganic and Theoretical Chemistry, University of Innsbruck, Austria

**Keywords:** CEC, Zwitterionic stationary phase, Natural products, Coffee

## Abstract

A methacrylate based monolith, containing the innovative zwitterionic monomer (3-allyl-1-imidazol) propane sulfonate, was prepared in 100 µm I.D. silica capillaries by UV initiated photo-polymerization. Composition of the porogen, i.e. a mixture of 1-propanol, 1,4 butanediol and water, was of great importance to obtain a homogeneous monolith with satisfactory permeability and good electrochromatographic performance. Morphology of the stationary phase was studied in Scanning Electron Microscopy and IR experiments, which revealed a good attachment to the capillary wall, flowthrough-pores in the range of 0.5–2 µm, and a continuous monolithic structure. The developed material was well suited for the analysis of six common phenolic acids (salicylic, cinnamic, syringic, rosmarinic, caffeic and chlorogenic acid) by CEC. Their separation was possible in less than 8 min with a mobile phase comprising a 12 mM aqueous ammonium acetate solution with pH 8.5 and acetonitrile, at an applied voltage of - 20 kV. The developed method was validated (R^2^ ≥ 0.995; LOD ≤ 3.9 µg mL^−1^, except for salicylic acid; recovery rates from 94 to 104%) and successfully used for the determination of phenolic acids in *Coffea arabica* samples. All of them contained cinnamic, syringic and caffeic acid, however only in unroasted coffee beans chlorogenic acid (0.06%) was found. The quantitative results were in good agreement to reported literature data.

## 1 Introduction

Despite the fact that Capillary Electrochromatography (CEC) is an attractive research area for academia, it is still barely relevant for daily life analysis. Many desirable features like high selectivity, versatility in respect to stationary phases and detection as well as an economic operation compete against not always applicable but severe disadvantages (limited reproducibility and robustness, availability of capillaries, etc.). Recent studies confirm the enormous innovative/resolution potential of CEC (e.g. monolithic phases prepared with ionic liquids [1] or decorated with graphene oxide [2], the option to use CEC-MS [3] or to separate enantiomers [4]). However, only practically relevant applications will promote the routine use of CEC. Currently their number is manageable, so that this technique is often considered as an exotic rather than an equivalent alternative.

When talking about challenging matrices for analysis natural products and their determination in biological samples (e.g. plants) are a good example. This is because their composition is usually complex, relevant compounds are sometimes present in small amounts, and they often show a high degree of structural similarity. A natural variation even within the same species, depending on factors like collecting season, soil or climatic conditions, further complicates their analysis. That CEC is suitable for the analysis of natural products has been shown in several publications, for example reporting on the determination of coumarins in *Angelica dahurica* [5], adrenergic amines in *Citrus aurantium* [6] or flavonoids in liquorice roots [7].

The preparation of monolithic phases is very variable; yet, of utmost importance are the monomers initially selected for polymerization. They determine polarity and selectivity of the material and have a significant impact on the generated electroosmotic flow (EOF) [8]. One interesting option is the use of zwitterionic monomers like SPE (*N*,*N*-dimethyl-*N*-methacryloxyethyl-*N*-(3-sulfopropyl) ammonium betaine) or MPC (2-methacryloyloxyethyl phosphorylcholine) [9,10]. The resulting phases show predominantly HILIC character, and they are especially suitable for the separation of polar constituents. The CEC use of such materials has been described [11–15]; however, their application to complex matrices is missing. This was our motivation to evaluate a newly developed monolithic zwitterionic CEC stationary phase for its potential to separate phenolic acids in coffee. Not only was the used monomer ((3-allyl-1-imidazol)propane sulfonate) unique, but also were monolith fabrication, characterisation and its application for quantitative analyses (including method validation) part of our investigations.

## 2 Experimental

### 2.1 Chemicals and standards

Standards of salicylic acid (**1**), cinnamic acid (**2**), syringic acid (**3**), rosmarinic acid (**4**), caffeic acid (**5**) and chlorogenic acid (**6**) were purchased from Sigma Aldrich (St. Louis, MO, USA); their purity was ≥98%. All solvents (acetonitrile, methanol) and chemicals (ammonium acetate and ammonia) required for CEC analysis had p.A. quality and were bought from Merck Eurolab (Vienna, Austria). Ultrapure water was prepared using a Sartorius arium purification system (Göttingen, Germany).

Chemicals for preparation of the monolith, i.e. 1,4 butanedione, 1-propanol, 2,2-diphenyl-1-picryl-hydrazyl, 3-(trimethoxysilyl) propyl methacrylate, dimethylformamide, allylimidazole, 1,3-propanesultone, dimethylformamide, 2,2-dimethoxy-2-phenylacetophenone and ethyleneglycol dimethacrylate were also obtained from Sigma Aldrich.

### 2.2 Preparation of monolith

For synthesis of the zwitterionic monomer 3-(1-allyl-1*H*-imidazolium-3-yl)propane-1-sulfonate 5.50 g allylimidazole (0.051 mM) were placed in 50 mL acetonitrile and 6.21 g 1,3-propanesultone (0.051 mM) were added at 0 °C. The mixture was kept under ultrasonic conditions for 2 h. A white solid precipitated, it was collected and dried under vacuum (yield: 60%; for IR and NMR spectra see supplementary information).

Prior to preparation of the monolith the silica capillary (100 μm I.D. with UV transparent coating; Polymicro Technologies, Phoenix, USA) was silanized following an already described procedure [16]. In brief, in a first step the capillary was etched with 0.1 M NaOH (100 °C for 1 h), rinsed with water, 0.1 M HCl and acetone and then dried in a stream of nitrogen. The silanization mixture (2,2-diphenyl-1-picryl-hydrazyl and 3-(trimethoxysilyl)propyl methacrylate in DMF) was filled in the capillary and the latter heated (120 °C) for 6 h. Then it was rinsed with DMF, acetone and dichloromethane and dried again.

A typical polymerization mixture consisting of 2% 2,2-dimethoxy-2-phenylacetophenone (w/w, with respect to the total mixture), 15% (w/w) (3-allyl-1-imidazol)propane sulfonate, 20% (w/w) ethyleneglycol dimethacrylate, 30% (w/w) 1-propanol, 30% (w/w) 1,4-butandiol and 15% (w/w) ultrapure water was prepared, and degassed by sonication for 5 min. The mixture was filled into silanized capillaries using a syringe and the latter sealed with silicon septa. The capillary was exposed to UV-light for 30 min using an 8 W, 254 nm lamp. Afterwards, the capillary was rinsed with acetonitrile for 3 h to remove the porogens and unreacted monomers. Before use the capillary was flushed with background electrolyte.

### 2.3 SEM and IR of monolith

Scanning Electron Microscopy (SEM) images of the monolith were recorded on a Jeol JSM-6010LV instrument (Tokyo, Japan) by applying an acceleration voltage of 10 kV. The sample was prepared by drying the capillary in an oven at 100 °C for 48 h, and cutting pieces of 2–3 mm with a razor blade. They were placed in a double sided carbon type sample holder and sputtered with gold prior to analysis. ATR-IR spectra were recorded on a Spectrum 100 (Perkin Elmer, Waltham, USA) equipped with a DicompTM crystal composed of a diamond ATR with ZnSe focusing element. The Spectrum software version 6.3.1.0134 was used for recording data. The background was taken using atmospheric conditions before measuring samples with 16 co added scans and a spectral resolution of 4 cm^−1^.

### 2.4 Sample preparation

Several *Coffea arabica* samples, either unroasted (UCB1, UCB2) or roasted (RCB1, RCB2), were purchased in local supermarkets in Innsbruck, Austria. The finely ground beans (250 mg) were extracted three times with 10 mL each of a methanol-water mixture (7:3) by sonication for 20 min. After centrifugation at 3200 rpm for 10 min (Hareus Labofuge 400, Langenselbold, Germany) the supernatant was combined in a round bottom flask and the solvent evaporated at 40 °C under reduced pressure. Prior to CEC analysis, the resulting residue was dissolved in 2 mL methanol and membrane filtered (0.45 μm ProFill cellulose syringe filters, Bruckner, Linz, Austria).

### 2.5 Analytical method

CEC experiments were performed on an HP^3D^ capillary electrophoresis system (Agilent, Waldbronn, Germany) equipped with Diode Array Detector, air-cooled column cartridge and automatic injector. The installed monolithic capillary column (total length 35 cm, effective length 27 cm, 100 µm I.D.) was coated with an UV-permeable polymer facilitating on-column detection at 280 nm. The optimum mobile phase (12% A/88% B) consisted of an aqueous 12 mM ammonium acetate solution with pH 8.5 (A; pH value adjusted with 3% ammonia) and acetonitrile (B). All buffers were membrane filtered (see above) prior to use and changed after three injections to assure reproducibility of the results. Separations were performed at an applied voltage of - 20 kV with inlet and outlet vials pressurized at 7 bar (N_2_) to prevent air-bubble formation. The separation temperature was 20 °C, and samples were injected in ‘high-flush mode’, i.e. by simultaneously applying 7 bar on the inlet vial and - 20 kV for a period of 3 s.

### 2.6 Method validation

The assay was validated according to ICH-guidelines and all respective data are summarized in Table 1. A stock solution was obtained by dissolving the standards in methanol, and further calibration levels were prepared by serial dilution in the ratio of 1:1 using the same solvent. LOD (S/N ratio 3) and LOQ (S/N ratio 10) values were visually evaluated from standard solutions. For determination of accuracy, sample UCB2 was spiked with three different concentrations (high, medium and low) of the standard compounds **1–6**. After extraction and CEC analysis the observed recovery rates were calculated by comparing the theoretical with the actually found concentrations. Intermediate precision was assured by determination of inter-day and intra-day variance of the results. For that reason, four portions of a sample (UCB2) were extracted and assayed under optimized conditions on three consecutive days; consistency of the results was evaluated based on peak area.

## 3 Results and discussion

### 3.1 Optimization of porogen composition

With weak hydrophobic and ion exchange properties the developed zwitterionic monolithic stationary phase exhibits mixed mode characteristics. It was prepared by in situ UV initiated polymerization of the monomer (3-allyl-1-imidazol)propane sulfonate and ethylene glycol dimethacrylate in presence of a ternary porogenic solvent. The choice of the latter plays an important role for the preparation of the monolith. It affects its porous structure, surface area, permeability and homogeneity. Typical binary porogenic mixtures commonly used for acrylate monoliths, such as cyclohexanol and decanol, toluene and dodecanol, or propanol and butanediol could not be utilized in the present work because of immiscibility with the zwitterionic monomer. Therefore, in order to overcome this problem, water was added to the binary porogenic solvents consisting of 1-propanol/1,4 butanediol to yield a homogeneous monolith exhibiting satisfactory permeability and good electrochromatographic performance. Methanol, as suggested in literature for the preparation of sulfoalkylbetaine/EDMA monoliths [10], was also tested as porogen; however, the obtained material was not sufficiently permeable.

### 3.2 Characterisation of monolith

**Fig. 1:**
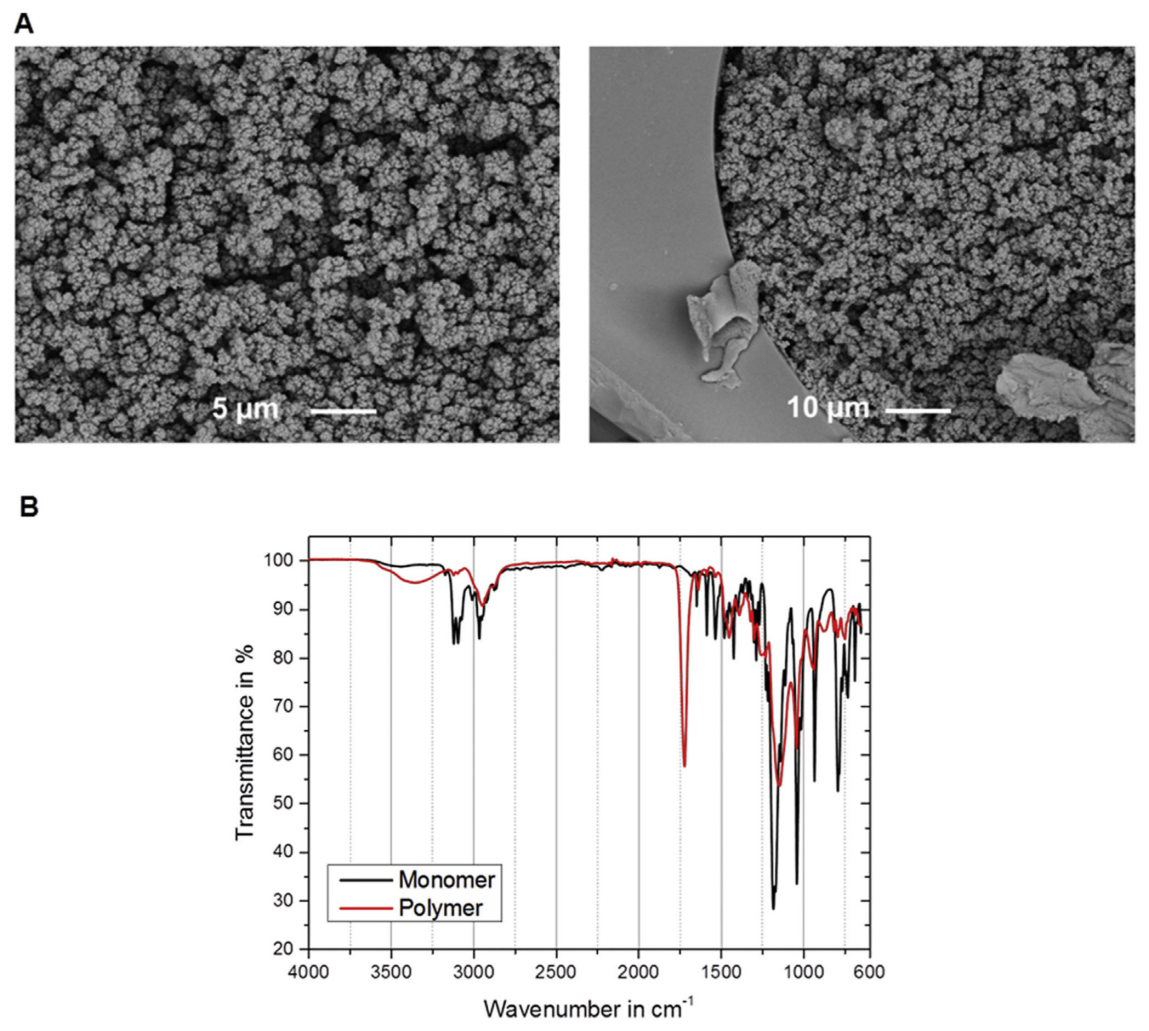
Characterisation of the developed monolithic material by electron microscopy (**A**; left: 1000-fold magnification, right: 500-fold magnification) and ATR-IR (**B**).

The morphology of the developed monolith was investigated using SEM and IR (Fig. 1). The first indicated a structure typical for a polymer monolith with clusters of homogeneous micro-globules and macroporous flowthrough-pores. In addition, the monolith was well attached to the inner wall of the fused silica capillary. The flowthrough-pores were in the range of 0.5–2 µm providing high permeability and low backpressure. ATR-IR spectra revealed that the previously very prominent vibrations from the ally group are no longer present in the polymer due to the polymerization reaction. The newly occurring strong absorption at 1720 cm^−1^ (ν C═O) indicates the presence of the crosslinker. Furthermore signals from the ${\text{SO}}_{3}^{-}$ group can be seen at 1146 cm^−1^ and 1042 cm^−1^. The C═N stretching vibration from the ring appears at 1630 cm^−1^. A series of signals can be found and assigned to the stretching vibrations of imidazole rings at 1422 cm^−1^, 1297 cm^−1^ and 1266 cm^−1^. Bending vibrations of the heterocycle occur at 932 cm^−1^, 793 cm^−1^ and 735 cm^−1^. Therefore one can conclude that the polymerization was successful and the monomers are linked to the crosslinker forming a continuous monolithic structure.

The EOF, which is responsible for generating the mobile phases flow, is caused by ionizable groups on the monoliths surface. Its amplitude is an indicator for the net surface charge density, and its prefix determines the direction of the EOF. The here described monolith includes sulfonic acid and imidazolium as ionic groups in addition to the hydrocarbon backbone, therefore an EOF in two directions is theoretically possible. For the developed monolith, both positive and negative groups of the zwitterionic monomer maintain their charge over the entire usable pH range.

The electrophoretic mobility of the newly developed monolith was determined using acetone as marker. An anodic EOF of about 4.95 × 10^−9^ m^2^V^−1^S^−1^ was obtained with a background electrolyte at pH 8.5. However, the effect of pH on the anodic EOF, which was studied between pH 6.5 (5.09 x 10^−9^ m^2^V^−1^S^−1^) and 10.5 (5.06 x 10^−9^ m^2^V^−1^S^−1^), was only moderate. On the contrary, the impact of acetonitrile concentration was more pronounced. It was investigated maintaining an ammonium acetate concentration of 12 mM and a pH of 8.5. The EOF was decreasing significantly by increasing the ACN concentration (70% ACN: 11.98 x 10^−9^ m^2^V^−1^S^−1^; 92% ACN: 3.58 x 10^−9^ m^2^V^−1^S^−1^), which can be attributed to a decrease in the dielectric constant and the magnitude of the zeta potential [17]. The effect of ionic strength of the background electrolyte on the EOF was studied by varying the concentration of ammonium acetate between 6 and 24 mM and keeping the ACN concentration at 88% and pH at 8.5. Within the tested range the anodic EOF decreased from 9.32 (6 mM) to 2.99 x 10^−9^ m^2^V^−1^S^−1^ (24 mM).

### 3.3 CEC method development

**Fig. 2:**
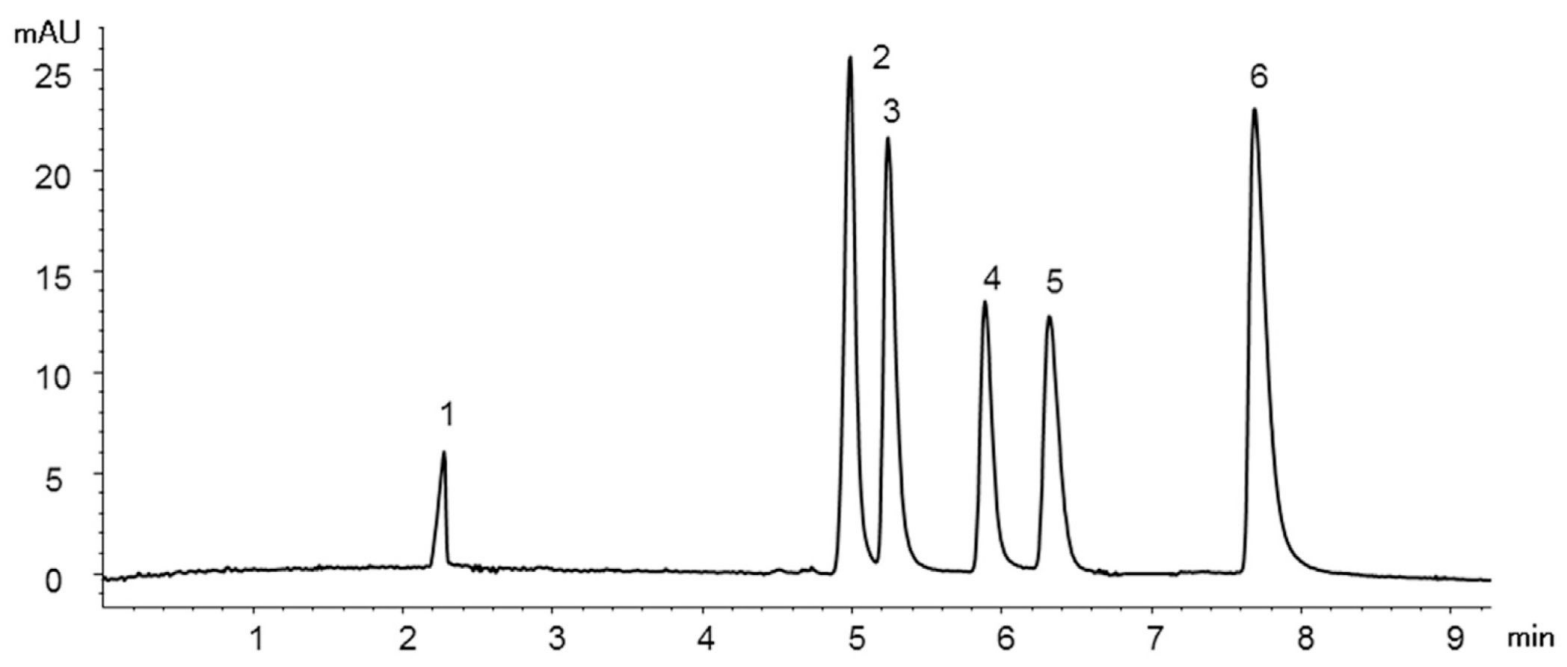
CEC separation of a standard mixture of six phenolic acids under optimized conditions. Peak assignment is according to Table 2.

The separation of a standard mixture of six organic acids (salicylic, syringic, cinnamic, rosmarinic, caffeic and chlorogenic acid) under optimized CEC conditions is shown in Fig. 2. Their baseline resolution was possible in less than 8 min. Several buffers and different instrumental settings were tested during method development. This revealed that higher percentages of water and an increased temperature were disadvantageous to generate a stable EOF, i.e. to prevent air bubble formation. It also was noticed that conditioning and proper flushing of the capillary are highly important. Accordingly, after each injection the capillary was flushed with the optimized buffer for 3 min by applying 7 bar (N_2_) at the inlet vial, and after the third injection both buffer vials were replaced with fresh ones.

**Fig. 3:**
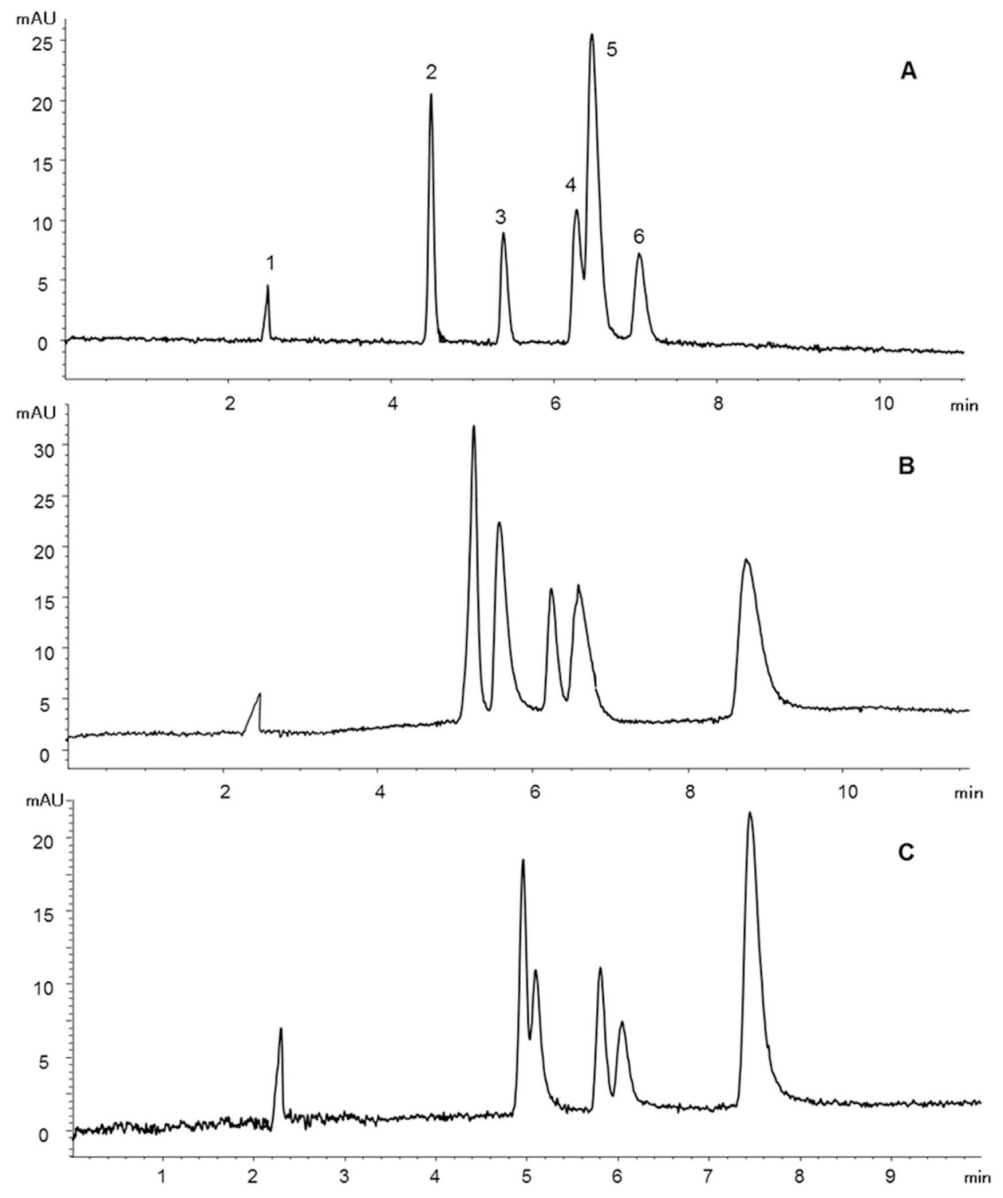
Influence of different parameters on the CEC separation of **1** to **6**, using a mobile phase with 82% acetonitrile (A), a molarity of 6 mM (B) or a pH-value of 9.5 (C); all other settings were optimal.

Mobile phase composition had the most pronounced effects on the results. For reversed phase type separations a higher organic modifier concentration usually decreases elution time and resolution also in CEC [18]. However, his effect might be reversed at very high levels of organic modifier. This seems to be valid for our experiments as well, because raising the percentage of acetonitrile in the buffer only by 4% (92 instead of 88%) resulted in an analysis time of 12 min and deteriorated peak shape. With 82% acetonitrile the run time was only slightly longer compared to the ideal buffer, but no separation of rosmarinic and caffeic acid was possible anymore (Fig. 3A). Concerning buffer molarity the typical range in CEC is 0.5–10 mM, and just like in CE higher values often result in prolonged analysis time due to a lower EOF [19]. For our application 12% aqueous 12 mM ammonium acetate solution in organic phase (equivalent to 1.6 mM in the buffer) turned out to be optimum, lowering the molarity to 6 mM (Fig. 3B) increased elution time and resulted in broader signals. Interestingly, at higher ionic strength (18 mM) retention time decreased, however cinnamic and syringic acid started to overlap. These unexpected observations might be due to the unique structure of the monolith, resulting in mixedmode separations combining characteristics of reversed phase and ion exchange type phases.

When optimizing buffer pH a value of 8.5 was found to be the optimum. The target analytes can be termed as strong (pk_a_ of salicylic acid: 2.75) to medium strong acids (pk_a_ of caffeic acid: 4.62) and predictions are difficult as a “nearly” non-aqueous buffer system was used for analysis. Yet, the selected value enabled faster and better separations compared to pH 9.5 (Fig. 3C) or pH 7.0. This might be explained by the fact that the charge of ionizable groups on the monolith is dependant on the pH of the mobile phase, thereby controlling the EOF. Varying the pH also resulted in profound effects on peak symmetry and shape.

The impact of temperature on the separation has already been mentioned and under the chosen conditions 20 °C were found to be the optimum. Above that an increased formation of air bubbles was observed, possibly caused by changes in buffer viscosity. This resulted in an instable current and a noisy baseline, an effect that could not be reverted by maintaining a pressure of 7 bar at the inlet and outlet vial during analysis. For migration of the target analytes it was required to apply negative voltage, otherwise no peaks were observed. Above the finally selected −20 kV the compounds eluted too quickly, so that they partially overlapped, at a lower voltage like −10 kV the runtime increased distinctively.

### 3.4 Method validation

Once the optimum CEC parameters were determined, the procedure was validated so that it could be used for the quantitative analysis of organic acids in *Coffea arabica* samples. For that purpose calibration curves of the six standard compounds were established. Depending on the type of compound the injected concentrations ranged from 2.4 mg mL^−1^ to 100 µg mL^−1^, and determination co-efficients higher than 0.995 indicate excellent linearity. LOD (S/N ratio 3) and LOQ (S/N ratio 10) values were determined from further diluted standard solutions and found to be lower than 3.9 and 11.7 µg mL^−1^ (both for compound **3**). Only for salicylic acid (**1**) the respective values were higher, which can be explained by a rather weak absorbance of this compound at the selected detection wavelength of 280 nm. Selectivity of the method was confirmed by two facts. First, in samples no impurities (shoulders) were visible in the relevant peaks, and second, their UV-vis spectra were identical to those of the standards and consistent as evaluated with the “peak purity option” in the operating software (Chemstation). For determination of accuracy, sample UCB2 was individually spiked with three different concentrations of all standard compounds. After extraction and analysis the observed recovery rates were found to range from 94% (**2**, low spike) to 104% (**3**, medium spike). Intermediate precision was assured by determination of inter-day and intra-day repeatability of the results. For that reason, four fractions of sample UCB2 were individually extracted and assayed under optimized CEC conditions three days consecutively. Variance of results within one day was always lower than 6.65% (compound **5**, day 2) and the maximum inter-day variance was 7.01% (**5**). Accordingly, all validation parameters (for details see Table 1) were satisfactorily met.

### 3.5 Analysis of samples

**Fig. 4:**
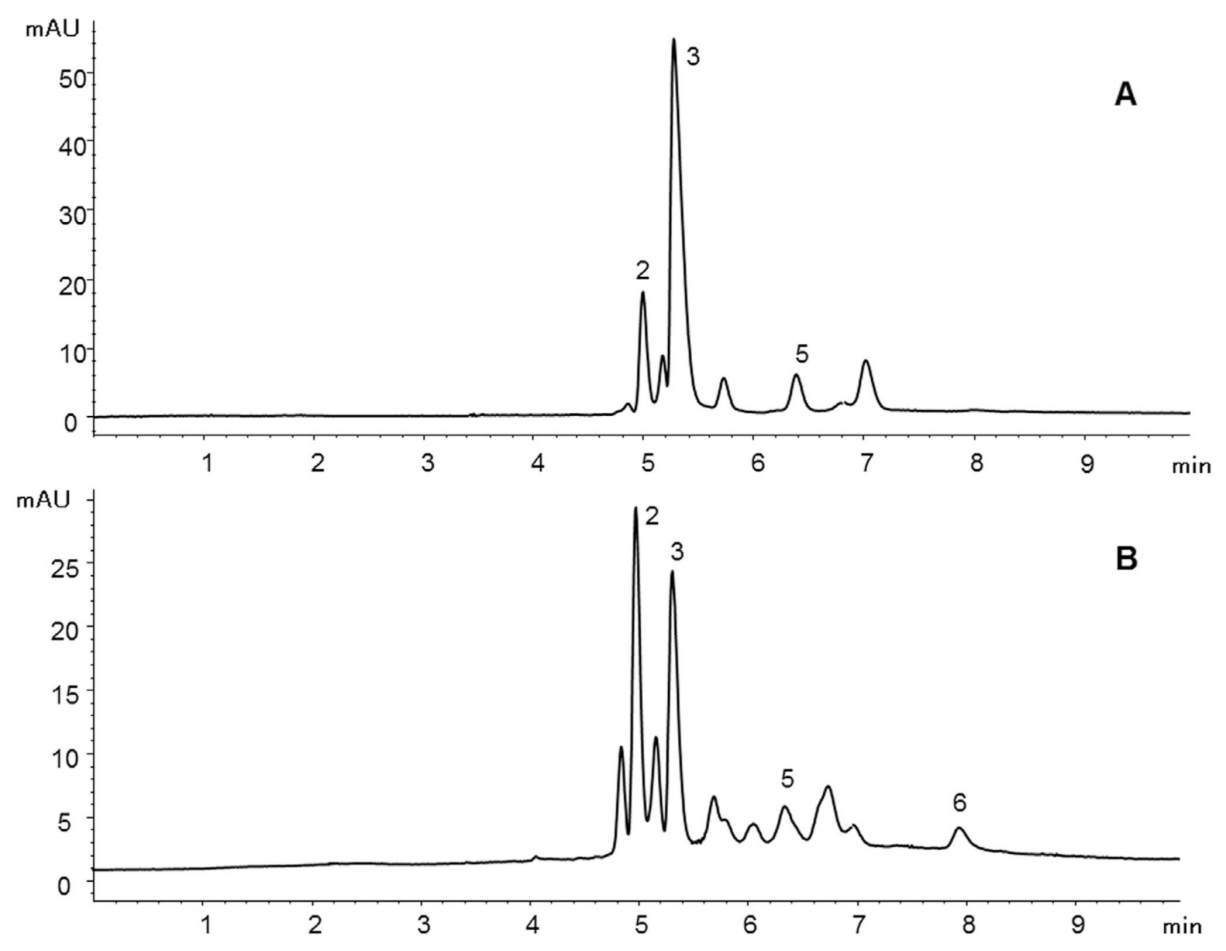
Separation of an unroasted (sample UCB2, A) and a roasted coffee bean sample (RCB1, B) under optimized CEC conditions. Peak assignment is according to Table 2.

The assignment of individual phenolic acids in different coffee bean extracts (see Fig. 4 for examples) was possible by matching retention times and UV spectra in comparison to the standards and by spiking with reference compounds. Table 2 lists the quantitative results of four samples. Compounds **1** and **4** could not be quantified in any of them, whereas compound **6** was detected only in samples RCB1 (0.061%) and RCB2 (0.064%). These values were in good agreement to published data which report that coffee might contain from 0.05% up to 1.70% of chlorogenic acid (**6**) [20]. Cinnamic and syringic acid were the most dominant acids in the specimens and they could be found in concentrations up to 0.18% (compound **3**). Caffeic acid (**5**) was present in all four samples ranging from 0.01% to 0.03%. Once again these results are similar to the outcome of another study in which a caffeic acid content of 0.06% in coffee beans is reported [21].

## 4 Conclusions

Even though Capillary Electrochromatography is still an interesting area of research, most of the published studies are dealing with the separation technique itself, with its principles, column technology or synthesis of stationary phases, rather than also applying the developed methods or materials on real-life analytical problems. In contrary, the here described innovative zwitterionic phase showed to be suitable for the reproducible, fast and accurate analysis of phenolic acids in coffee bean extracts. A comparison of our assay with already published ones for the determination of phenolic compounds results in the following conclusions. Naturally, its sensitivity and separation efficiency cannot compete with UHPLC-MS based approaches, which report LOD values in the low ng mL^−1^ range [22] or enable the identification of close to 50 compounds in 11 min [23]. However, compared to more related studies our method is at least equivalent, as for example the CEC separation of five catechins and xanthines in tea required more than 25 min [24], and LOD values for phenolic acids ranged from 1.5 to 2.5 µg mL^−1^ [25]. Also performance characteristics were similar (e.g. repeatability for the quantitative determination of amines in bitter orange by CEC ≤4.1%; [6]), so that distinct features of the here described procedure are definitely innovativeness of the zwitterionic monomer used, practical confirmation of the proposed concept including monolith fabrication and application, as well as compliance of the assay with validation criteria. Fundamental studies like the here presented one might “only” open the door for further investigations, but hopefully they spark interest in CEC, alleviating its transition from a technique of mainly academic interest to one with practical relevance.

## Supplementary Material

**Appendix A. Supplementary data**

Supplementary data related to this article can be found at http://dx.doi.org/10.1016/j.aca.2017.01.048.

supplementary

## Figures and Tables

**Table 1:** Performance characteristics of the developed CEC assay. Assignment of compounds is according to Table 2.

Compounds						

Parameter	1	2	3	4	5	6
Regr. Equation	y=0.033x−5.266	y=0.652x+15.896	y=0.469x+4.398	y=0.115x+8.115	y=0.366x−7.331	y=0.196x+20.000
R^2^	0.9921	0.9968	0.9976	0.9951	0.9986	0.9958
Range^a^	2451−153.0	506−31.6	898−56.1	1083−67.7	986−61.6	2054−64.2
LOD^a^	18.5	2.1	1.4	3.9	1.9	3.6
LOQ^a^	55.5	6.3	4.2	11.7	5.7	10.8
Precision						
intra-day^b^	–	5.65	6.47	–	6.55	–
inter-day^c^	–	4.59	1.71	–	7.01	–
Accuracy^d^						
High spike	98	96	97	97	104	94
Medium spike	102	102	104	95	96	95
Low spike	101	94	96	103	98	101

a μg mL^−1^.

b Maximum deviation within one day based on peak area in percent.

c Deviation over three days based on peak area in percent.

d Expressed as recovery rates in percent.

**Table 2:** Quantitative determination (weight percent) of phenolic acids in different coffee bean extracts with relative standard deviation in parentheses (n=3)

	Compounds/sample	UCB1	UCB2	RCB1	RCB2
**1**	Salicylic acid	–	–	–	–
**2**	Cinnamic acid	0.028 (3.64)	0.049 (2.93)	0.028 (2.22)	0.045 (3.14)
**3**	Syringic acid	0.185 (4.46)	0.067 (3.56)	0.082 (3.40)	0.069 (2.88)
**4**	Rosmarinic acid	–	–	–	–
**5**	Caffeic acid	0.027 (4.60)	0.014 (2.64)	0.023 (4.49)	0.015 (1.58)
**6**	Chlorogenic acid	–	–	0.061 (4.66)	0.064 (4.32)

## Highlights

- The fabrication and characterisation of an innovative zwitterionic monolith.
- CEC method development and validation.
- Quantitative application of the monolithic material for natural products analysis.
