# Supplementary material for: An innovative monolithic zwitterionic stationary phase for the separation of phenolic acids in coffee bean extracts by capillary electrochromatography
Source: Anal Chim Acta. Author manuscript; Available in PMC 2017 Apr 22. (PMC5388181; doi:10.1016/j.aca.2017.01.048)

**An innovative zwitterionic stationary phase for the separation of phenolic acids in coffee bean extracts by capillary electrochromatography**

**SUPPLEMENTARY INFORMATION**

Adele Murauer^1^, Rania Bakry^2^, Herwig Schottenberger^3^, Christian Huck^2^, Markus Ganzera^1^

^1^ Institute of Pharmacy, Pharmacognosy, University of Innsbruck, Innsbruck, Austria

^2^ Institute for Analytical Chemistry and Radiochemistry, University of Innsbruck, Innsbruck, Austria

^3^ Institute of General, Inorganic and Theoretical Chemistry, University of Innsbruck, Austria

Characterisation of (3-allyl-1-imidazol)propane sulfonate

Melting point: 159-161°C

IR (ATR): 3138, 3080, 3054, 2973, 1686, 1594, 1451, 1422, 1274, 1179, 1123, 1030, 995, 937, 886, 800, 771, 626, 613, 601, 568, 521, 446

^1^H NMR (300 MHz, MeOH): δ 9.07 (I=1, t), 7.75 (I=1, t), 7.63 (I=1, t), 6.10 (I=1, m), 5.46 (I=1, dxd), 5.42 (I=1, m), 4.89 (I=2, d), 4.46 (I=2, t), 2.83 (I=2, t), 2.32 (I=1, t)

^13^C NMR (75 MHz, MeOH): δ 137.83, 132.24, 124.12, 123.94, 122.00, 52.97, 49,50, 48.68, 27.30

For respective spectra (IR & NMR) see next pages.

^1^H NMR spectra of (3-allyl-1-imidazol)propane sulfonate


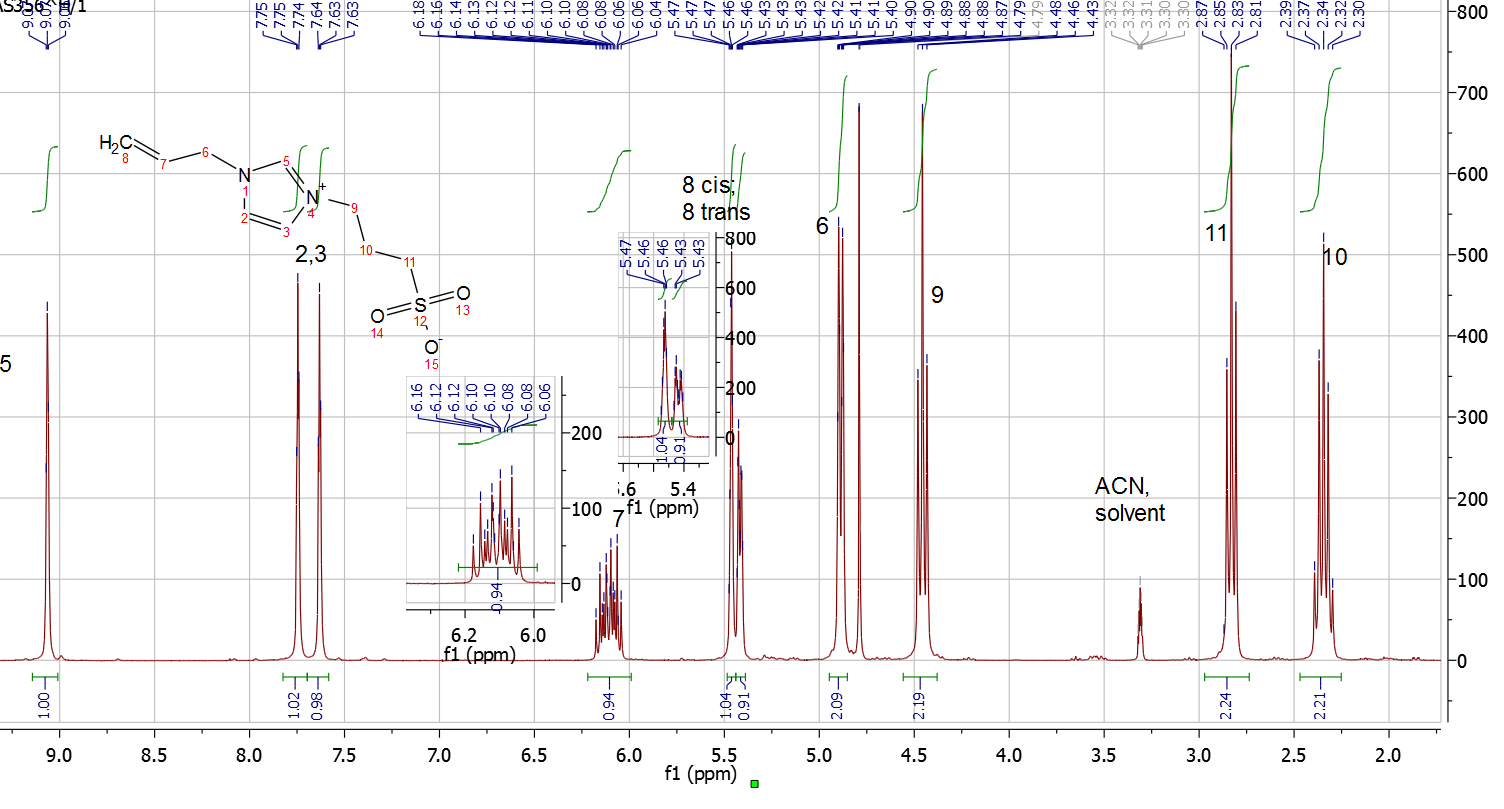


^13^C NMR spectra of (3-allyl-1-imidazol)propane sulfonate


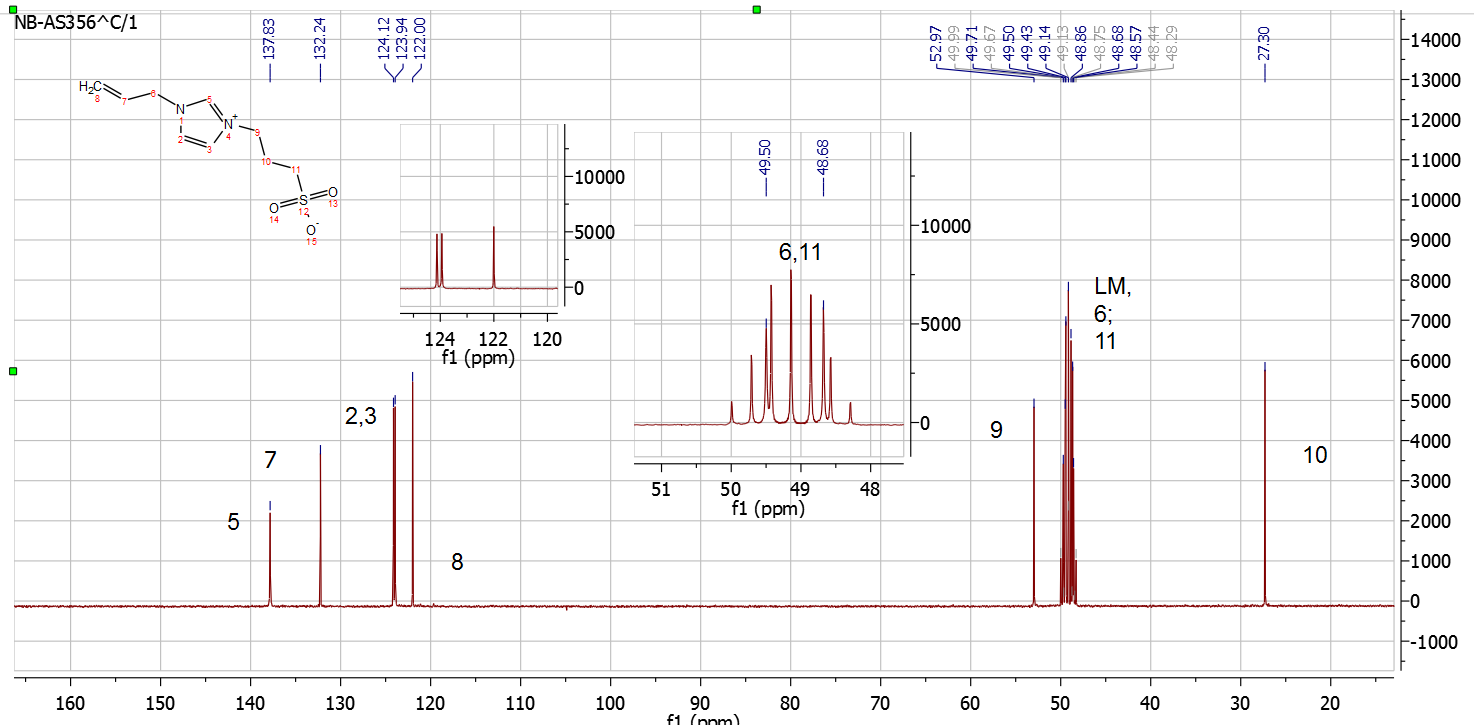


IR spectra of (3-allyl-1-imidazol)propane sulfonate


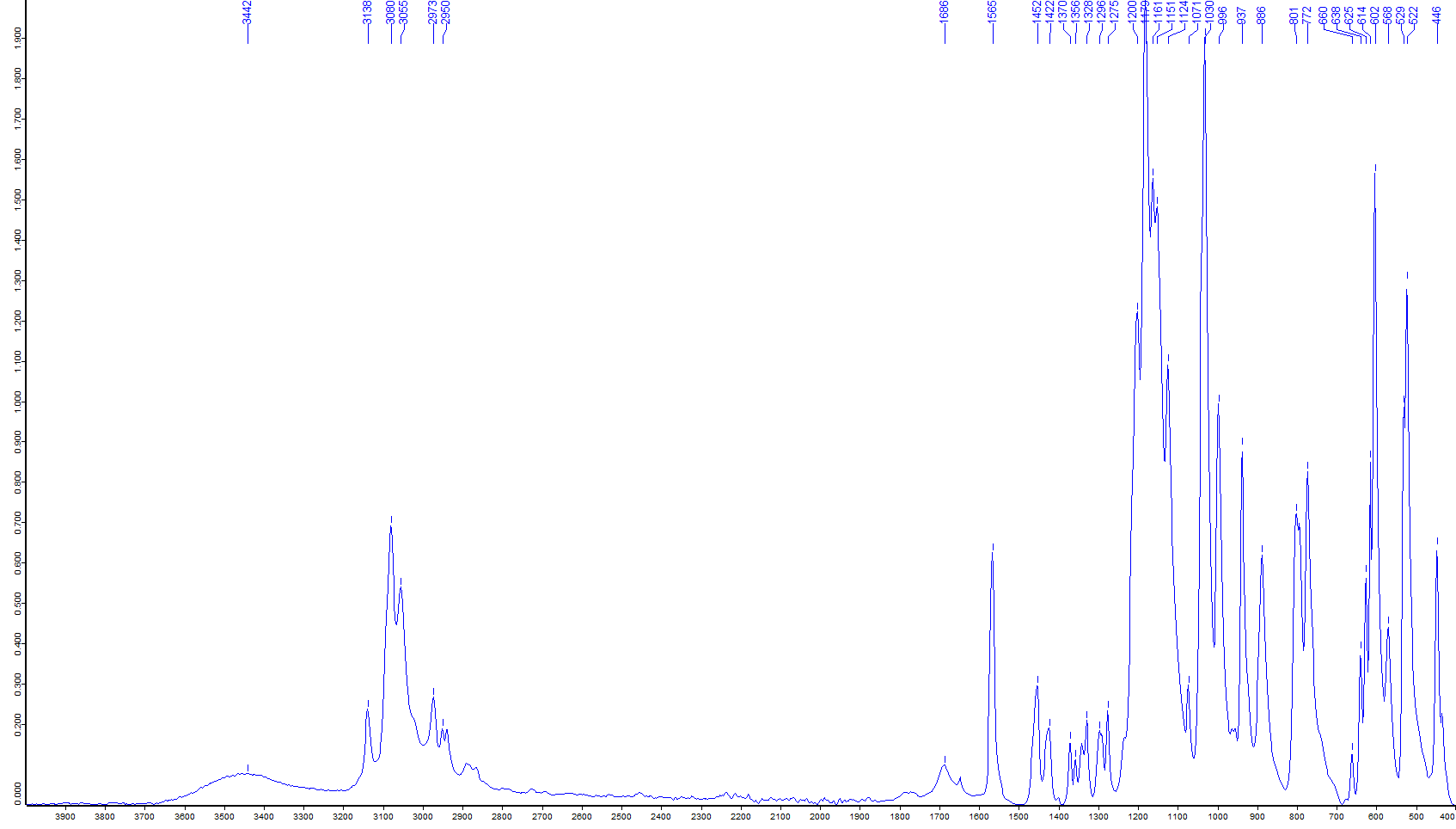

Supplement: supplementary [file NIHMS72231-supplement-supplementary.docx]
